# *In Vitro* and *In Vivo* Characterization of a Typical and a High Pathogenic Bovine Viral Diarrhea Virus Type II Strains

**DOI:** 10.3389/fvets.2018.00075

**Published:** 2018-04-13

**Authors:** Dario Amilcar Malacari, Andrea Pécora, Maria Sol Pérez Aguirreburualde, Nancy Patricia Cardoso, Anselmo Carlos Odeón, Alejandra Victoria Capozzo

**Affiliations:** ^1^Instituto Nacional de Tecnología Agropecuaria (INTA) – Instituto de Virología, Centro de Investigaciones en Ciencias Veterinarias y Agronómicas, Buenos Aires, Argentina; ^2^Instituto Nacional de Tecnología Agropecuaria (INTA) – Instituto de Patobiología, Centro de Investigaciones en Ciencias Veterinarias y Agronómicas, Buenos Aires, Argentina; ^3^Consejo Nacional de Investigaciones Científicas y Técnicas, CONICET, Buenos Aires, Argentina; ^4^Instituto Nacional de Tecnología Agropecuaria (INTA) – Estación Experimental Agropecuaria Balcarce, Buenos Aires, Argentina

**Keywords:** bovine viral diarrhea virus, virulence, colostrum-deprived calves, apoptosis, immune-suppression

## Abstract

Non-cytopathic (ncp) type 2 bovine viral diarrhea virus (BVDV-2) is widely prevalent in Argentina causing high mortality rates in cattle herds. In this study, we characterized an Argentinean ncp BVDV-2 field isolate (98-124) compared to a high-virulence reference strain (NY-93), using *in silico* analysis, *in vitro* assays, and *in vivo* infections of colostrum-deprived calves (CDC) to compare pathogenic characters and virulence. *In vitro* infection of bovine peripheral blood mononuclear cells (PBMC) with BVDV 98-124 induced necrosis shortly after infection while NY-93 strain increased the apoptotic rate in infected cells. Experimental infection of CDC (*n* = 4 each) with these strains caused an enteric syndrome. High pyrexia was detected in both groups. Viremia and shedding were more prolonged in the CDC infected with the NY-93 strain. In addition, NY-93 infection elicited a severe lymphopenia that lasted for 14 days, whereas 98-124 strain reduced the leukocyte counts for 5 days. All infected animals had a diminished lymphoproliferation activity in response to a mitogen. Neutralizing and anti-NS3 antibodies were detected 3 weeks after infection in all infected calves. Virulence was associated with a more severe clinical score, prolonged immune-suppression, and a greater window for transmission. Studies of apoptosis/necrosis performed after *in vitro* PBMC infection also revealed differences between both strains that might be correlated to the *in vivo* pathogenesis. Our results identified 98-124 as a low-virulence strain.

## Introduction

Bovine viral diarrhea virus (BVDV) is a common pathogen of ruminants worldwide. Infections with BVDV lead to significant economic losses and have an important impact on the cattle industry (1–6). By infecting cells of the immune system, BVDV evokes an immunosuppression leading to a decreased immune response to other infectious agents (7). The immunosuppression elicited by BVDV includes a transient leukopenia in calves (8, 9), with diminished counts in peripheral B and T lymphocytes (10, 11), as well as monocytopenia and neutropenia (11, 12) with functional impairment of monocytes, dendritic cells, neutrophils, and lymphocytes (9, 11, 13, 14). BVDV also causes persistent infections (PI) by infecting the fetus in the early gestation period. Once colostrum-derived immunity has declined, these PI animals continuously shed BVDV in large quantities (15, 16).

Bovine viral diarrhea virus is characterized by a strong genetic diversity and can be divided into two main species, BVDV-1 and BVDV-2, each subdivided into sub-types (17). According to their effect on cell culture, two biotypes have been described, cytopathic (cp) and non-cytopathic (ncp), being ncp BVDV more frequent in nature. BVDV type-2 has been related with severe acute clinical signs and hemorrhagic disorders (18). In Argentina, ncp BVDV-2 strains have been associated with acute hemorrhagic enteritis in young cattle (19). BVDV-2 strains can be classified as high virulent or low virulent based on *in vivo* observations, such as pyrexia (i.e., T° >40°C for high virulent strains) and the time span of lymphopenia, among other signs (20). The characterization of the virulence of different BVDV isolates was also determined by the clinical onset in newborn calves, as a model of infection (9, 12, 20–23). Many BVDV strains have been evaluated using colostrum-deprived calves (CDC) (20). This animal model is more suitable for reproducing the disease under controlled conditions, due to the lack of any maternal immune component that may interfere with virus infectivity.

Two nucleotide substitutions within the 5′ untranslated region (UTR) have been described, which differed between high- and low-virulence BVDV-2 isolates (24). These changes, positioned within the predicted IRES, may modulate the virus replication rate. However, later studies revealed that the correlation between sequence motifs and virulence are unclear and thus, other factors apart from sequence analysis should be considered (25).

*In vitro* studies have shown to be useful to study virulence of field strains. The reduction in the proliferation of lymphoma BL-3 cells by *in vitro* infection with BVDV-2 strains has been applied as a qualitative measure of the lympho-cytopathogenicity induced by the virus (26). This fact can be used to estimate the percentage of apoptotic and necrotic circulating lymphoid cells after *ex vivo* BVDV-2 infection and as a parameter to define the degree of virulence of BVDV-2 strains.

The aim of this study was to characterize an Argentinean BVDV-2 ncp isolate, associated with mild enteritis and general dermatitis in the field. Virulence analysis of this strain was performed in parallel with a previously characterized high virulent type 2 strain isolated in New York in 1993 “NY-93” (26, 27) using *in silico* and *in vitro* methods, supported by *in vivo* infection of CDC. We used virus grown in MDBK cells, as Meyer et al. (28) demonstrated that MDBK-grown NY-93 strain, even produced by a cDNA clone, was clinically indistinguishable from the wild-type virus in animal experiments. We also evaluated if the clinical signs developed by the CDC in response to the experimental infection with BVDV 98-124 were similar to those described in the field. This study can be helpful to further assess therapeutic treatments and vaccines against BVD.

## Materials and Methods

### Viruses

Non-cytopathic BVDV 98-124 strain (genotype 2b, GenBank accession number MH074881) was isolated from tissue samples of cattle that showed purulent nasal secretions, ptyalism, severe hemorrhagic watery diarrhea, and dehydration, during an outbreak in Buenos Aires province (Argentina) in 1998 (29). Details of the necropsy findings during this outbreak were reported elsewhere (19). The virus used in this study was obtained from peripheral blood mononuclear cells (PBMC) of an infected calf, processed for virus isolation (VI) by inoculating a 10% cell homogenate onto cultures of Madin–Darby Bovine kidney (MDBK) cells, as described below. Stock was prepared from a third passage in MDBK cells.

The BVDV-2 strain New York 93 (NY-93) was isolated in 1993 from an outbreak that took place in New York following the importation of a heifer from Canada. The severe acute clinical presentation included hemorrhagic signs, elevated rectal temperatures and death loss (30). The NY-93 BVDV-2 strain, kindly provided by Dr. R. Donis, was used as a “control reference” virus strain in this work.

### Animals

Newborn male Holstein calves were purchased from a dairy farm in Buenos Aires. They were removed from their mothers immediately after birth before they can consume colostrum. Calves (mean weight at birth 32.13 ± 2.03 kg) were housed in the biosecurity boxes (BSL2) located at the Research Center of Veterinary Sciences (CICVyA) INTA Castelar, Buenos Aires. They were negative for BVDV at birth, as determined by VI from WBC samples followed by detection based on reverse transcriptase polymerase chain reaction (RT-PCR) assay (31). They were also free for antibodies against BVDV at birth, as assessed by serum neutralization test using BVDV type-1 (NADL strain) and BVDV type-2 VS253 strains (29).

Upon arrival, calves were sanitized with 0.2% benzalkonium chloride and dried with a towel with vigorous movements to stimulate the peripheral circulation. The navel was disinfected with iodine alcohol (1:1 alcohol and iodine) twice a day until it was dry. An antibiotic-therapy schedule was applied: Ceftiofur Sodium (Ceftiomax, Biogenesis Bagó, Argentina) was administered intramuscularly on daily bases during the first 10 days of life (dose: 6 mg/kg of body weight) and Gentamicin sulfate was injected intravenously once a day during the first 5 days of life (Gentamicin, Equi Systems, Argentina; 8 mg/kg of body weight).

Calves were fed with a milk replacer (AF80, ACA, Argentina), free of immunological components and balanced food (18% protein content; Start calves, ACA, Argentina). The milk replacer was always administered warm (37–38°C) using a calf feeding bottle and getting the calf in a head up position to obtain a proper esophageal leak enclosure. They were periodically assessed for serum neutralizing antibodies (Nab) against BVDV type-1 and type-2, as described above. All animals received 1 ml of vitamin AD3E (500,000 UI Vitamin A, Vitamin D3 75,000 UI, Vitamin E 50 mg; Bagó AD3E, Biogenesis, Argentina) parenterally.

Colostrum-deprived calve handling, inoculation, and sample collection were performed by trained personnel under the supervision of a veterinarian and following national animal welfare regulations (Protocol N° 02/2010 from CICUAE, INTA).

### Sequence Analysis of the 5′UTR Region

Polymerase chain reaction and sequencing for 98-124 strain was performed as previously described (29). Sequences were trimmed and analyzed with Bioedit Software (32) to obtain a 236 bp fragment, corresponding to the 5′UTR region of BVDV. A multiple sequence alignment of four low-virulence strains (17011, 7937, 713, and 5521; GenBank accession numbers AF039179, AF039175, AF039177, and AF039174, respectively), four high-virulence strains (890, NY-93, 17583, and 23025; GenBank accession numbers AF039180, AF039173, AF039176, and AF039172, respectively), Osloss reference strain (M96687) and 98-124 strain (MH074881) was performed with ClustalW, Version 1.8.3 (33). Analysis of virulence indicators considered nucleotide positions 219 and 278 of Osloss strain as described by Topliff and Kelling (24).

### Apoptosis–Necrosis Assessment

Peripheral blood mononuclear cell (1 × 10^6^ per well) were infected with 98-124 isolate (multiplicity of infection “mock” = 1) or mock infected (cells incubated with a clarified MDBK lysate instead of viral inoculum) for 1 h at 37°C in duplicates, washed by centrifugation and cultured in complete RPMI (containing 10% BVDV-free FCS, already controlled for BVDV antigen, genome and antibodies; 1X Glutamine; 25 mM HEPES and 1X Antibiotic-Antimycotic, all reagents are from GIBCO^TM^) in a 48-well plate at 37°C and 5.5% CO_2_. The cells were analyzed by flow cytometry using PE Annexin V Apoptosis Detection Kit (BD Biosciences Pharmingen, CA, USA) as described before (9). Annexin V and 7-AAD negative are viable cells. Early apoptotic cells are Annexin V positive and 7-AAD negative while late apoptotic and necrotic cells are 7-AAD positive (34).

### Experimental Infection

Eight animals were randomly assigned to two treatment groups (*n* = 4 animals each) and infected at 50 days of age (mean weight 55.93 ± 2.15 kg) with 5 ml of a culture supernatant containing 10^6^ TCID_50_/ml of ncp BVDV 98-124 or ncp BVDV NY-93 strain. Virus inoculum was instilled into the nostrils. Infections with the two different strains were performed 4 months apart in order to prevent possible co-infections.

### Monitoring of Clinical Signs and Sampling

All inoculated calves were examined twice a day. Rectal temperature and clinical signs were recorded. Based on the observation of anorexia, sensorium, diarrhea, nasal and ocular discharge, and respiratory signs (dyspnea and cough) a clinical score was designed (Table 1). The recorded values for each 98-124 or NY-93 infected CDC (*n* = 4 for each group) were plotted and computed as the area under the curve (AUC). Whole blood samples and nasal swabs (NSs) were collected 1 day before challenge and daily during the first 20 days post-infection (dpi). Blood cells were separated by and put through one freeze–thaw cycle for virus isolation. NSs were immediately submerged in 1 ml of DMEM cell culture medium and placed on ice. Blood samples were also collected in heparinized sample tubes for leukocyte counts and ARN extraction, these samples were processed in an automatic hematology analyzer Celldyn 3500-LASER (Abbott Laboratories, Abbott Park, Ill, USA).

**Table 1:** Clinical signs and designated score.

Clinical Sign	Observed symptom	Score
Sensorium	Alertness	0
	Depressed	1
	Obtunded	2

Anorexia	Absent	0
	Present	1

Nasal Discharge	Serous	1
	Seromucosal	2
	Mucopurulent	3

Ocular Discharge	Serous	1
	Seromucosal	2

Diarrhea	Mild	1
	Moderate	2
	Watery	3
	Hematochezia	4

Respiratory Signs or changes in the respiratory pattern	Cough	1
	Dyspnea	2

### Virus Isolation

Thirty microliters of lysate [either white blood cells (WBC) or NS] were used to inoculate 4 wells of a 96-well plate with 70% of confluent MDBK cells. After rocking at 37°C for 1 h, the inoculum was removed from the cells and replaced with 150 µl of cell culture media. After 3 days, the cell culture (including media) was frozen at −80°C. After thawing to room temperature, 30 µl of the resulting lysate was added to a fresh MDBK cells monolayer and rocking at 37°C for 1 h, after that the inoculum was replaced with 150 µl of cell culture media. After three passages in MDBK cells, detection of BVDV antigens was performed by IF (VMRD BVDV Direct FA Conjugate, WA, USA).

### Humoral Immune Response

A commercial kit was used to detect antibodies against BVDV NS3 following manufacturer’s instructions (PrioCHECK™ Bovine BVDV Ab Plate Kit, Thermo Fisher Scientific, USA). Nab titers in serum samples were tested by virus-neutralization against the homologous strain used for each experimental infection, as described previously (29).

### Proliferation Inhibition Assay

Peripheral blood mononuclear cells from infected animals with BVDV 98-124 and NY-93 strains were stimulated with Concavalin A (ConA, Sigma, USA). Inhibition of PBMC proliferation was evaluated at 0, 5, 8, 10, and 18 dpi. Briefly, triplicates of PBMC cells were seeded in 96-well plates (300,000 cells/well) and incubated with ConA (10 µg/ml) in 100 µl cell culture media for 72 h at 37°C in an atmosphere of 5% CO_2_. After the incubation, the proliferative response was determined by using the XTT assay following the manufacturer’s instructions (TACSTM XTT Cell proliferation/viability assay, R&D Systems, MN, USA). Results were expressed as the percentage of residual proliferation respect to values obtained at 0 dpi.

### Quantitative Real-Time Polimerase Chain Reaction (qRT-PCR)

Virus RT-qPCR was performed using Pan Pestivirus primers 324-326 (2) to amplify a 288 pb fragment of the 5′UTR region of BVDV. RNA was extracted from blood and frozen NS of infected animals and from infected PBMC and cell culture media of the apoptotic–necrotic assay using Trizol Reagent (Invitrogen, CA, USA). Reverse transcription was carried out using the antisense primer and Reverse Transcriptase (from Moloney murine leukemia virus [M-MLV]; Promega, USA) under standard conditions and 500 ηg of total ARN. The EVA Green^®^ dye was used in this real-time RT-PCR method contained in the master mix (Mezcla Real; Biodynamics, Argentina) and ROX reference dye (Invitrogen, USA). The standard samples used were plasmid containing BVDV PCR products cloned into pGemT (Promega, WI, USA). Samples and standards were run in triplicate in an ABI 7500 thermocycler (Applied Biosystems, CA, USA) and analyzed using model 7500 SDS software v 1.3.1.

### Statistics

Neutralizing antibody titers were logarithmically transformed (log10) to comply with homogeneity of variance and normal distribution. In those cases when the data met a normal distribution (Wilk–Shapiro normality test) and their variances were comparable, Bartlett Test statistical analyzes were performed using one- or two-way ANOVA followed by Bonferroni multiple comparison test, using STATISTIX 7x software (Analytical Software, TN, USA). For comparing clinical AUC scores of two groups of data, the Mann–Whitney test was used. The analysis was performed with MedCalc version 12 (Medcalc bvba software used, CA, USA). In all cases, a confidence interval of 95% was considered.

## Results

### Analysis Sequence Motifs in the 5′UTR Region

**Figure 1:**
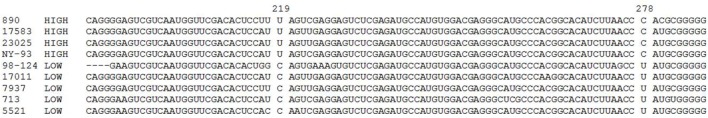
Type 2 bovine viral diarrhea virus 5′UTR sequence alignment. Squares show nucleotide differences at positions 219 and 278 between low- and high-virulence strains. Accession numbers: 23025: AF039172, NY-93: AF039173, 5521: AF039174, 7937: AF039175, 17583: AF039176, 713: AF039177, 17011: AF039179, 890: AF039180; 98-124: MH074881.

The sequences obtained by RT-PCR amplification and direct sequencing of NY-93 and 98-124 strains were trimmed and analyzed. The two-base substitution described for BVDV-2 strains (24) were mapped along the sequence comprised between positions 219 and 278 of the 5′UTR. The low-virulence strain (98-124) presented a Cytosine at position 219, while the high-virulence strain (NY-93) had an Uracil. On the contrary, at position 278, the high-virulence strain had a Cytosine whereas the low-virulence strain had an Uracil (Figure 1).

### *In Vitro* Studies

**Figure 2:**
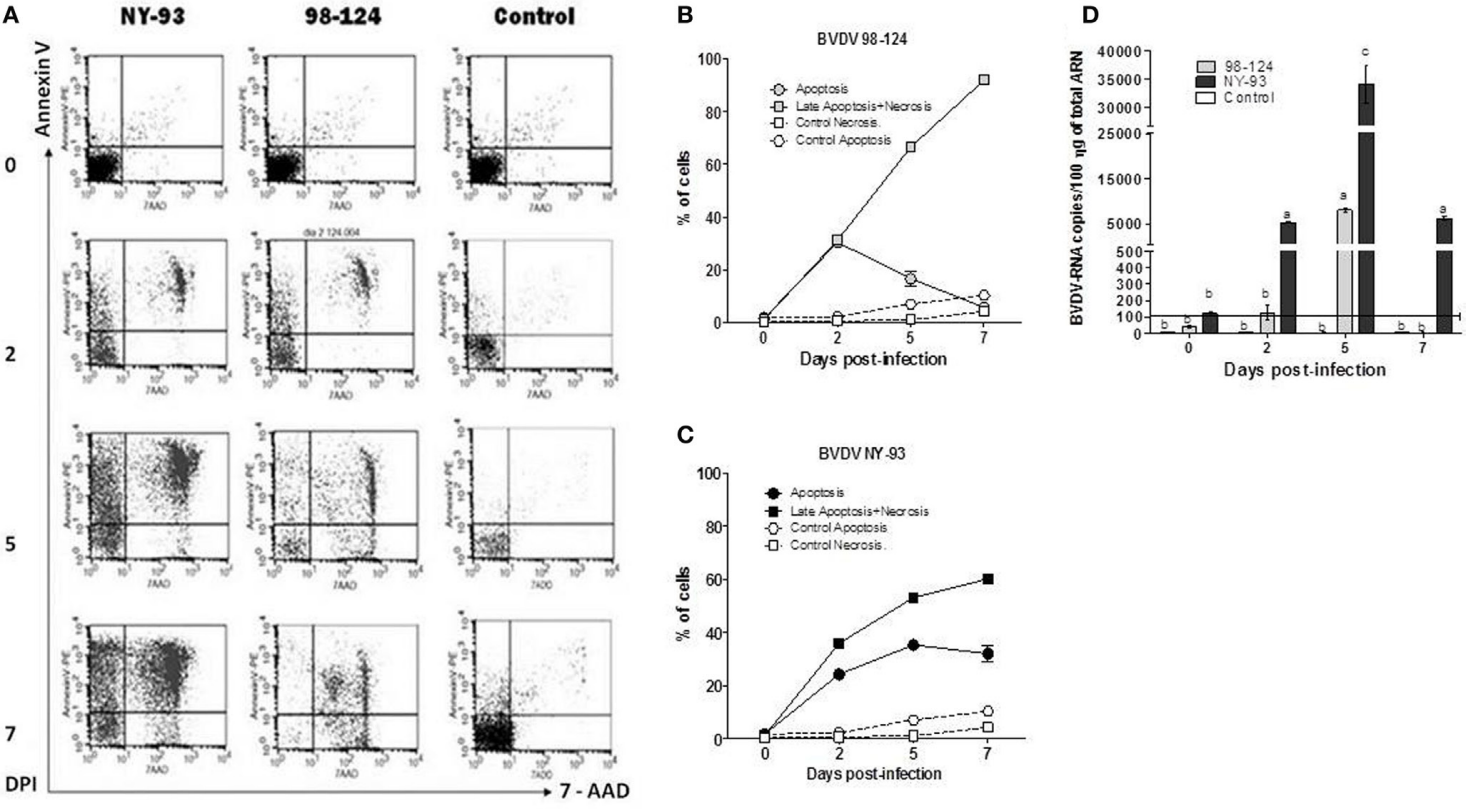
Viability and virus load of *ex vivo*-infected peripheral blood mononuclear cells (PBMC). PBMC were infected with bovine viral diarrhea virus (BVDV) NY-93 or 98-124 strains and stained with Annexin V/7-AAD at 0, 2, 5, and 7 DPI. **(A)** Dot plot of a representative experiment showing apoptotic and necrotic cells at different days after infection. Line graphs depict the kinetics of apoptotic and necrotic PBMC infected with 98-124 **(B)** and NY-93 **(C)**. **(D)** Viral RNA load in infected PBMC. Results are expressed as RNA viral copies/100 ng of total RNA. Results are mean values from three independent experiments. Horizontal line indicates the sensitivity limit of the qRT-PCR. DPI, days post infection. Lowercase letters above each bar indicate significant differences (*p* < 0.05).

Peripheral blood mononuclear cells (PBMC) from BVDV-negative calves were infected *in vitro* with both type-2 strains (moi = 1) and the viability of the infected cells was assessed by measuring apoptosis and necrosis using Annexin V/7-AAD staining. The basal rate of apoptotic and necrotic cells was 1.77 and 1.22%, respectively. PBMC *in vitro* infected with 98-124 underwent cell death shortly after infection. The number of dead cells displayed a maximum rate of 91.9% at 7 dpi (Figures 2A,B). A shift from 7-AAD negative to positive staining in Annexin V positive cells (upper left to right area) was observed over time for PBMC infected with NY-93 strain. Apoptotic cells (Annexin V positive, 7-AAD negative) peaked at 5 dpi (34.15%) and decreased thereafter up to 29.89% at 7 dpi, while dead cells (both necrotic or late apoptotic) increase from 36.02% at 2 dpi to 60.3% at the end of the experiment (Figure 2C).

A maximum peak in viral RNA levels was detected at 5 dpi (8,400 copies per 100 ηg of total RNA) in peripheral blood mononuclear cells (PBMC) infected with BVDV 98-124. Values decreased thereafter to non-detectable levels at 7 dpi. Conversely, PBMC infected with NY-93 showed a sharp increase in the number of viral RNA copies at 2 dpi that peaked at 5 dpi, with values approximately 3.5 times higher than those observed with the other strain (*p* < 0.05; Figure 2D). Similar levels of RNA to those measured at 2 dpi we still observed at 7 dpi. At all-time points, RNA levels were higher in PBMC infected with NY-93 compared to BVDV 98-124.

### Experimental Infection of CDC

Groups of 4 CDC were infected, with 98-124 or NY-93 strain, at 50 days of age when immune parameters were comparable to those described for adult animals. Clinical signs and rectal temperature were recorded twice a day and clinical score values were plotted and computed as the AUC. The assigned clinical score for each clinical sign was detailed in Table 1.

**Figure 3:**
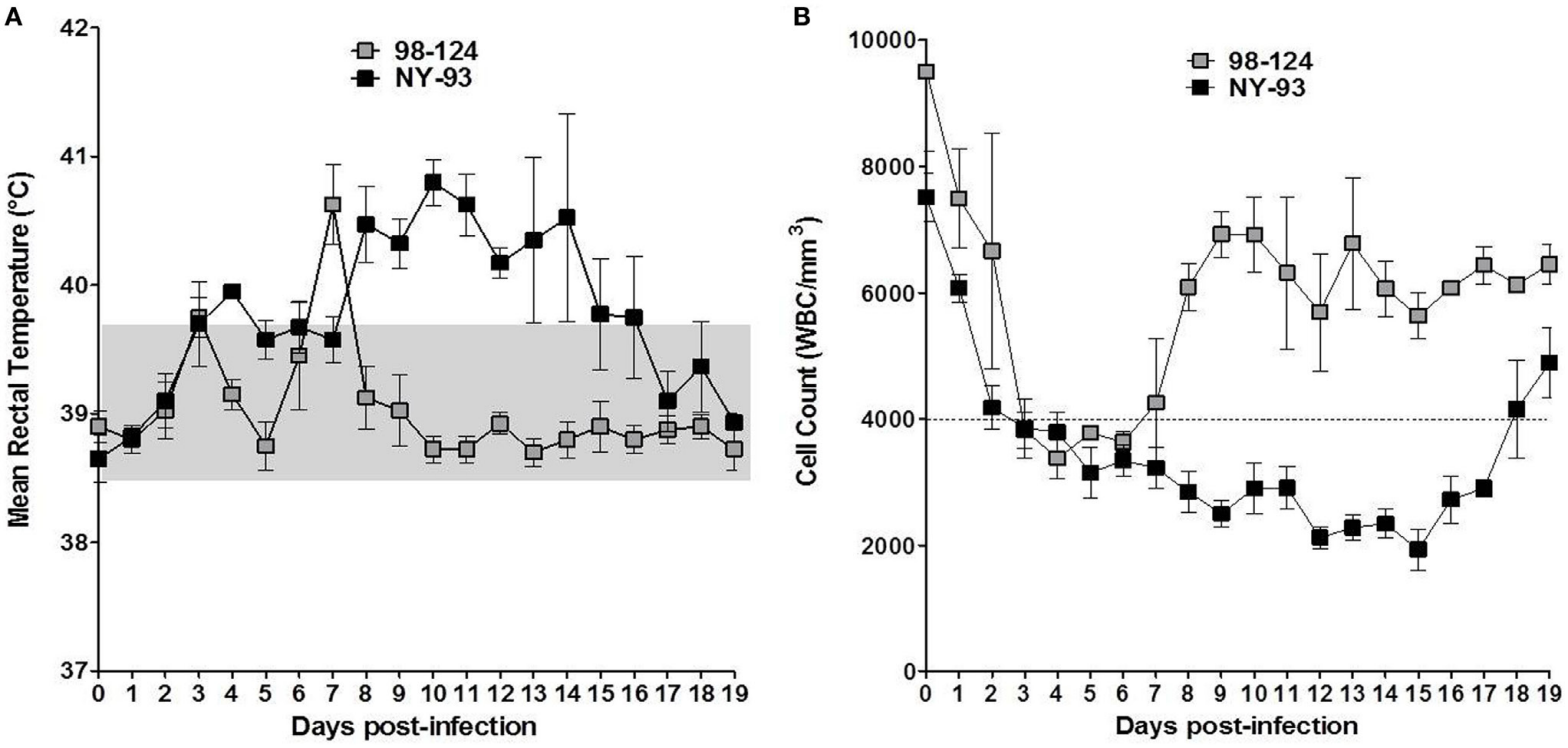
Clinical signs in infected calves: temperature and lymphopenia. Kinetics of mean rectal temperatures **(A)** and white blood cells (WBC) counts **(B)** measured in colostrum-deprived calves infected with bovine viral diarrhea virus 98-124 or NY-93 strains at different days post-infection. The dotted line indicates the lower limit of WBC count to consider a clinical leukopenia and the gray square delimits the normal rectal temperature range for calves.

All challenged calves developed gastrointestinal signs after BVDV infection. Clinical signs were mild in the group infected with BVDV 98-124. Calves exhibited depressed sensorium associated with pyrexia and anorexia. A biphasic pyrexia profile was found around 3 dpi and between 6 and 8 dpi (Figure 3A). A non-aqueous mild diarrhea developed after infection that continued for 2 days. All animals showed a mild hematochezia during the first day of the diarrhea. The AUC for the group infected with 98-124 was 10 ± 2.16, mainly due to the presence of diarrhea.

Calves infected with NY-93 developed anorexia and depressed sensorium from 4 to 18 dpi. They also underwent hemostatic disorders evidenced by a decreased clotting time (data not shown) and hematochezia. Calves suffered from aqueous diarrhea and a marked dehydration (>5%, from 11 to 16 dpi) and received proper rehydration treatment. Only two animals infected with NY-93 strain presented biphasic pyrexia, with the first peak between 3 and 6 dpi and a maximum record of 39.9°C; while the other peak was observed between 7 and 18 dpi (42.1°C). The other two animals showed sustained pyrexia from 2 to 12 dpi and from 1 to 18 dpi. The maximum *t*° was 41.2°C (Figure 3A). The AUC for the NY-93 CDC infected was 37.75 ± 8.07. This value was significantly higher than that scored by BVDV 98-124 (*p* < 0.05).

Animals infected with 98-124 strain developed leukopenia between 2 and 7 dpi with a minimum count of 2520 L/mm3, while leukopenia was recorded between 2 and 19 dpi in animals infected with NY-93 with a minimum count of 1280 L/mm3 (Figure 3B). The average WBC reduction was about 68 and 78% for 98-124 and NY-93 strain infected group, respectively.

**Figure 4:**
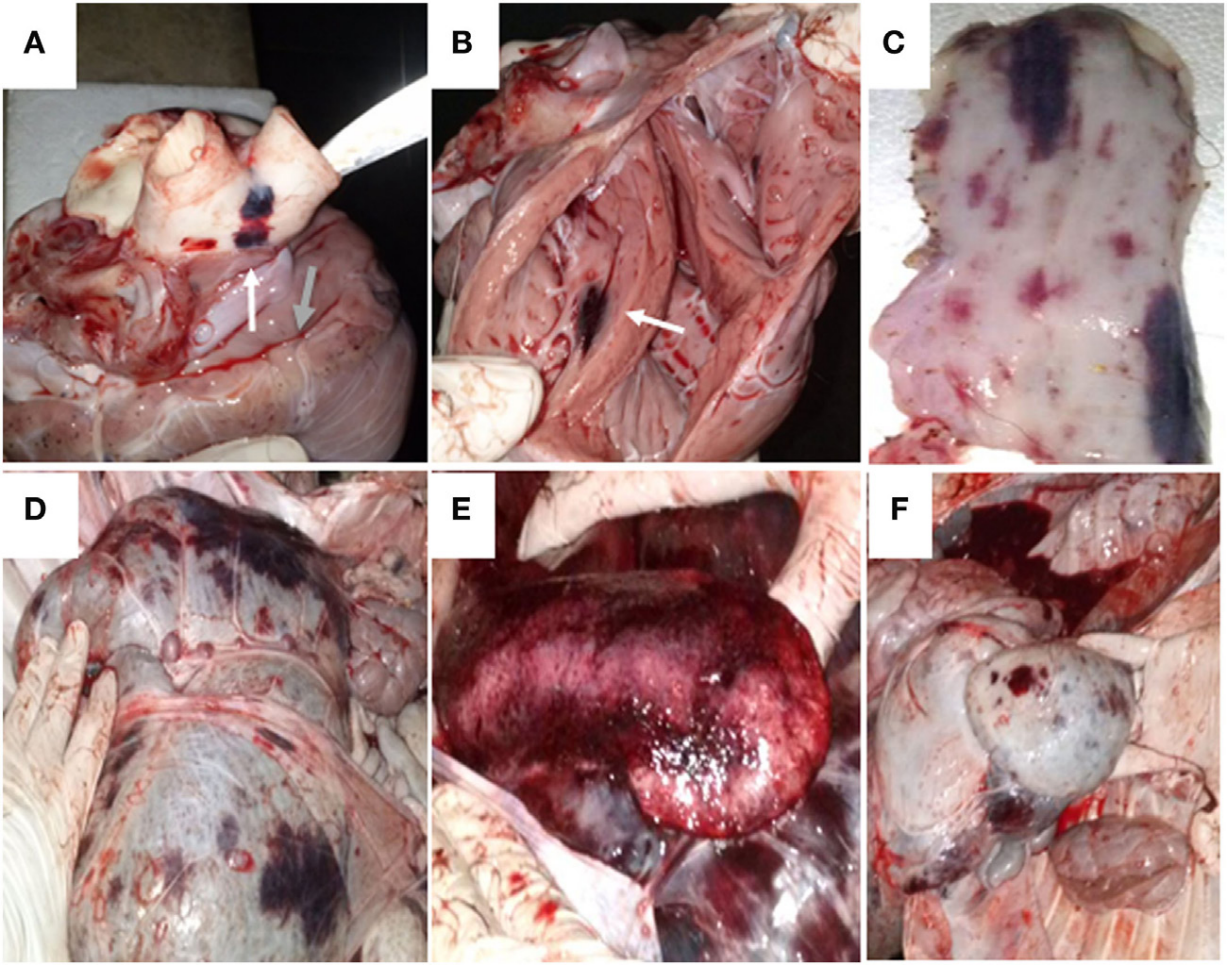
Necropsy of the animal infected with bovine viral diarrhea virus NY-93. The animal was found dead on day 18 post infection. **(A)** The yellow arrow indicates a disseminated bleeding in the base of the aorta and pulmonary arteries. The green arrow shows petechiae in the pericardium. **(B)** The white arrow depicts bleeding in the endocardium. Disseminated bleeding was found in esophagus **(C)**, rumen **(D)**, spleen **(E)**, and reticulum **(F)**.

One animal infected with NY-93 strain died at 18 dpi, even though it received intra-venous fluids. It had interdigital hemorrhagic ulcerations in the right forelimb and in the ocular membrane, and petechiae with widespread distribution in the lungs and pleura. Blood vessels were enlarged and the heart showed signs of endocarditis. The abdominal cavity and the gastrointestinal track also had the same pattern of lesions, with widespread petechial hemorrhages in several tissues, including the mucosa (Figure 4).

### Viremia and Shedding

**Figure 5:**
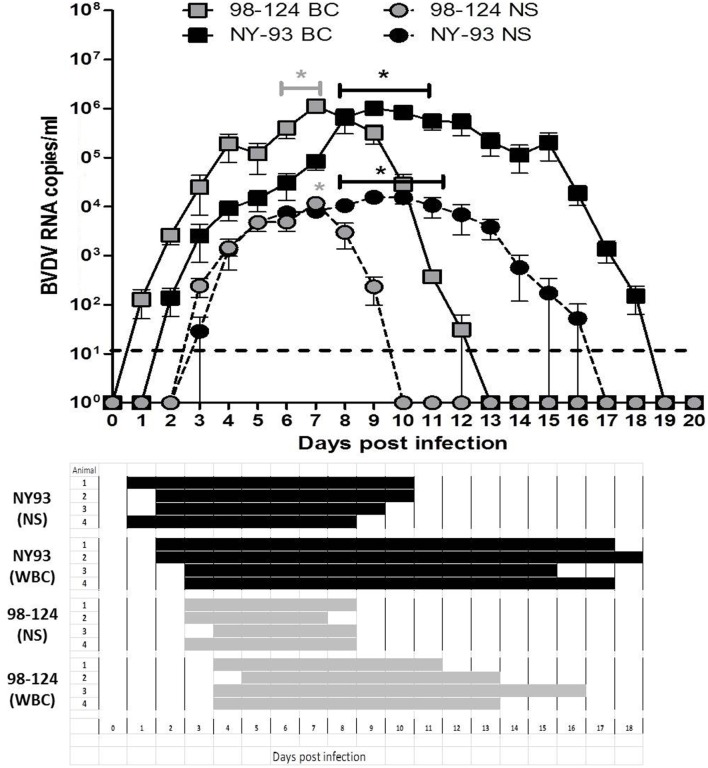
Viremia and shedding. Upper panel: bovine viral diarrhea virus (BVDV) RNA quantification (qRT-PCR) from blood or nasal swabs (NSs) samples of colostrum-deprived calves (CDC) infected with either BVDV strain at different times post infection. Results are expressed as viral RNA copy number per milliliter. (*) values significantly superior to those measured at 0 dpi (*p* < 0.05). Lower panel: viral isolation from white blood cells (WBC) and NSs of CDC infected with both BVDV strains.

Viral RNA was detected in blood from 1 to 12 dpi in calves infected with 98-124 strain, with a maximum number of viral RNA copies at 7 dpi (1.1 × 10^6^ ± 3.2 × 10^5^ copies/ml). Animals infected with NY-93 strain had positive viral RNA detection from 2 to 18 dpi, with a maximum values measured from days 8 to 10 post-infection (Figure 5, upper panel). Virus was isolated from WBC at 4 dpi in BVDV 98-124 infected calves, and viral detection was positive for about 10 days. Virus isolation results were positive for NY-93 infected animals from days 3 to 17 post-infection (Figure 5, lower panel).

Viral RNA was detected in NS samples from 3 to 9 dpi in all animals infected with 98-124 with a peak value of 1.1 × 10^4^ ± 5.4 × 10^3^ copies/ml at 7 dpi. Animals infected with NY-93 had positive shedding for 14 days (3–16 dpi) and the mean maximum viral RNA load was ~8 × 10^3^ ± 3 × 10^3^ copies/ml from 8 to 11 dpi (Figure 5, upper panel). Virus was isolated from nasal secretions from 3 to 8 dpi in calves infected with 98-124 strain; and from 2 to 10 dpi in those infected with NY-93 (Figure 5, lower panel).

### Effect on Adaptive Immunity

**Figure 6:**
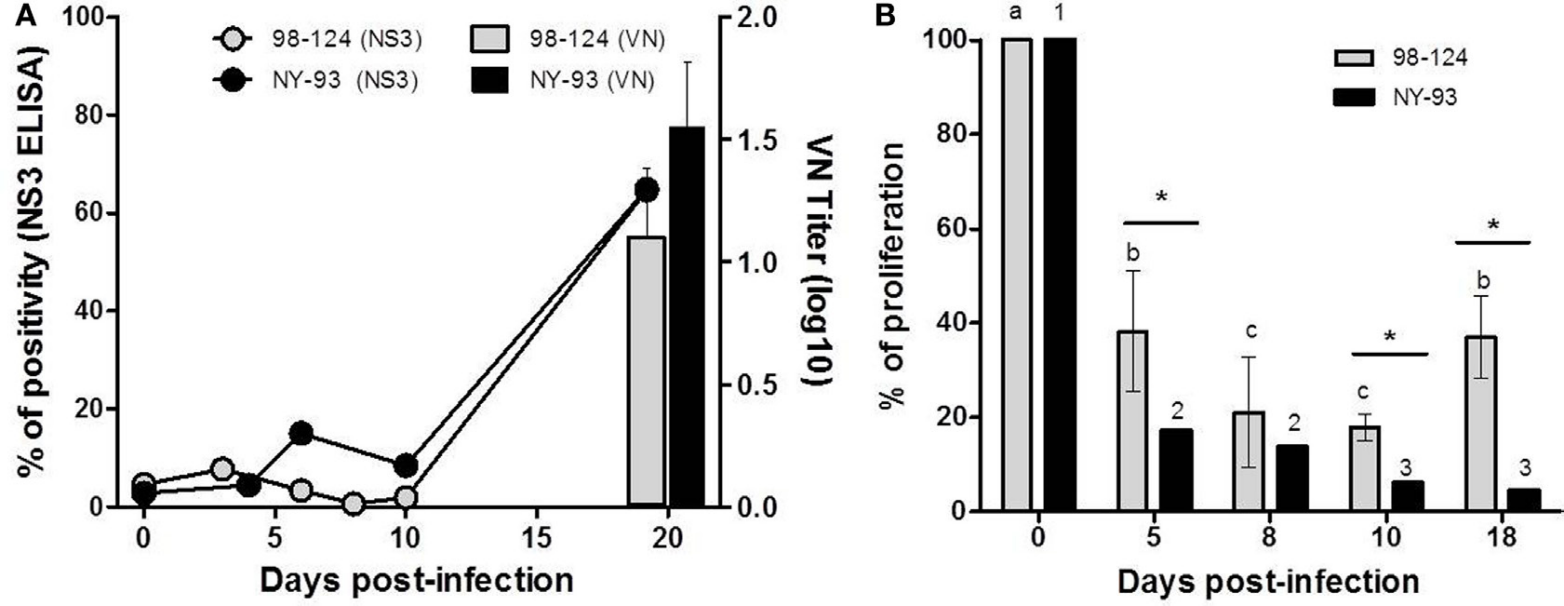
Humoral responses and impairment of cell-mediated immunity. **(A)** Humoral response of colostrum-deprived calves infected with bovine viral diarrhea virus 98-124 or NY-93 strains. Connecting lines correspond to antibodies against NS3 and neutralizing antibodies against each homologous strain are shown with bars. **(B)** Cellular immune response was tested by a proliferation assay performed using peripheral blood mononuclear cells (PBMC) from calves at different days post infection and compared to values obtained at 0 dpi. Lowercase letters and numbers above each bar indicate significant differences (*p* < 0.05) between time points for 98-124 or NY-93 infected PBMC, respectively. (*) Significant differences between groups are indicated (*p* < 0.05).

Neutralizing antibodies against each homologous strain as well as antibodies against NS3 were assessed in serum samples obtained at 0 dpi and after infection, up to 20 dpi. In both experimental groups, neutralizing and anti-NS3 antibodies were first detected at 20 dpi (Figure 6A).

Peripheral blood mononuclear cells from all infected calves had a reduced capacity to proliferate in response to Concanavalin A (ConA) from 5 to 18 dpi. The maximum reduction in the proliferation index in PBMC obtained from BVDV 98-124 infected CDC was observed at 8 and 10 dpi, compared to values measured at 0 dpi. The capacity of these cells to respond to ConA stimulation was partially recovered at 18 dpi, with similar values to those found at 5 dpi (*p* < 0.05; Figure 6B). Proliferation capacity was reduced gradually over time, and the lowest proliferation rate was measured at 10 and 18 dpi (Figure 6B).

## Discussion

We have characterized an Argentinean BVDV type 2 strain in terms of virulence and pathogenicity, using *in silico, in vitro*, and *in vivo* methods. The BVDV-2b 98-124 Argentinean isolate had *in silico, in vitro*, and *in vivo* features to classify it as a typical low-virulence type 2 strain. The parallel evaluation of the highly virulent and well-characterized NY-93 reference strain reinforced our experimental approach.

*In silico* analysis involved the evaluation of previously described nucleotide virulence indicators (24). We found a correlation between the presence of an Uracil and a Cytosine residues in positions 219 and 278 of the 5′UTR, respectively; and the clinical presentation of the disease in the CDC. Based on these markers, BVDV 98-124 was classified as a low-virulence strain. Although sequence analysis is fast to perform and easy to analyze, some flows have been reported. Giangaspero and Harasawa (35) showed that the substitution described by Topliff and Kelling (24) was only observed in some strains of genotype 2b-BVDV, and that two hyper-virulent strains isolated from sheep had cytosine and Uracil in positions corresponding to that of the low-virulence strains. The authors highlighted the need to considering evolutionary divergence between strains when searching for common features as indicators of virulence. Thus, more studies are needed to apply sequence analysis for characterizing BVDV-2 strains. In fact, Tautz et al. (25) reported that there is no clear correlation between specific sequence motifs and virulence, and proposed to analyze virulence considering multiple factors.

By performing *ex vivo* infections of bovines PBMC, we verified that virus load was higher and lasted longer in PBMC infected with NY-93, compared to 98-124. We also studied apoptosis and necrosis rates in these infected cells. Lymphocyte apoptosis has been previously described after BVDV infection (12). Apoptosis and necrosis rates of lymphocytes and lymphoid cell lines have been associated with virulence of BVDV strains (7, 26). Here, we showed that *in vitro* PBMC infection with both BVDV strains revealed a different rate of apoptosis and necrosis. BVDV 98-124 induced a rapid increase in the number of necrotic cells; conversely, NY-93 induced an elevated apoptotic rate over time, with cells moving from being Annexin V positive and 7-AAD negative toward double positives over time. This pattern is usually observed for truly apoptotic cells.

The high virulent strain seems to be acting as a less acute “killer,” promoting a high apoptotic rate sustained over time. The interpretation on the role of apoptosis and necrosis of PBMC in the pathogenicity of several viruses including BVDV has changed over the last 10 years. Multiple forms of regulated necrosis have shown to have a key role in pathologies such as sepsis, inflammatory diseases and infectious disorders (36). A burst of necrotic bodies shortly after infection may trigger a pro-inflammatory response that can promote, anti-viral responses and secretion of TNF-α among other pro-inflammatory cytokines. The pattern of apoptosis/necrosis induction may be regulated by TNF-α while the production of TNF-α can be associated with BVDV virulence (37). Further experiments are needed to clarify the relationship between TNF-α and BVDV-2 virulence and the possibility of assessing virulence by measuring apoptosis rates of *in vitro* infected PBMC.

Leukopenia is a clinical sign associated with the infection of cattle with different strains of BVDV (11, 37). Experimental infection of CDC with both the high and low virulence provoked a reduction in WBC counts greater than 60% for both strains; however, leukopenia differed in the duration, suggesting that NY-93 would expose infected animals to acquire secondary infections for longer periods than the low-virulence strain. Another interesting observation is the comparable level of reduction in WBC counts, suggesting that this value by itself may not be a feature strictly related to the virulence of the infecting strain. Thus, it is important to consider other clinical signs such as rectal temperature and clinical signs, as discussed below.

We observed biphasic pyrexia as the infection proceeded. This pattern has already been reported in CDC (12, 23, 38). The severity of pyrexia after infection has been associated with the virulence of the strain, which is concordant with our results.

Both groups developed a gastrointestinal syndrome with watery diarrhea and depression after infection, with differences in the severity and duration of the symptoms. Calves infected with BVDV 98-124 suffered a mild non-watery diarrhea that lasted for one or two consecutive days, while those infected with NY-93 developed a severe gastrointestinal syndrome with watery diarrhea and depression for several consecutive days. One of the four NY-93-infected calves died at 18 dpi, caused by the hemostatic disorder. This type of hemorrhagic syndrome has been previously described in experimental infections with this particular strain and with other BVDV-2 strains (27, 39).

The severity of clinical signs can be related to the level of viremia (40). Calves infected with BVDV 98-124 or NY-93 showed differences in the time-span of viremia (circulating virus) and shedding (virus in NSs), while peak amounts of BVDV RNA in both blood and NSs were comparable. In concordance with previous observations, in our experimental conditions and based on the significantly longer period of viral excretion detected, the high-virulence NY-93 strain would be more effective in the transmission by aerosol or nasal contact than 98-124.

Another effect of BVDV on the immune system is the interference with cell-mediated immunity *in vivo*. Here, we showed that infection of CDC with both strains impaired lymphoproliferation in response to a mitogen along all the observation period. Interestingly, the lymphoproliferation capacity was only partially recovered at 18 dpi only in 98-124 infected calves. These results evidenced the immune suppression triggered by BVDV infection that accounts for the susceptibility usually observed against opportunistic pathogens. These results also show that this effect seems to be sustained in time for the highly virulent strains.

The presence of Nab in animals infected with BVDV has been usually detected approximately 3 weeks after challenge (41). In this study, CDC infected with both strains developed antibodies against NS3 and low titers of Nab at 20 dpi, consequent with the immunosuppression caused by these ncp BVDV strains.

We have classified the Argentinean field isolate BVDV 98-124 as a low virulent strain by assessing different parameters both *in vitro* and *in vivo*. The use of a well-characterized strain was a valuable tool to validate our results, supported by the fact that the strain produced on MBDK cells is relevant for an *in vivo* study (28). *In silico, in vitro* and *in vivo* assessments were concordant in identifying high and low-virulence strains. *In vitro* studies involving PBMC infection followed by assessment of apoptosis and necrosis rates may be explored as possible indicators of BVDV-2 virulence.

## Ethics Statement

Animal handling, inoculation, and sample collection were performed by trained personnel under the supervision of a veterinarian and following national animal welfare regulations (Protocol No. 02/2010 from CICUAE, INTA).

## Author Contributions

DM carried out most of the experiments as part of his PhD thesis, with the collaboration of AP and MA. DM and NC wrote the manuscript with support from AC, MA, and AP. NC helped supervise the project. AO and AC conceived the original idea, procured funding, and supervised the project.

## Conflict of Interest Statement

The authors declare that the research was conducted in the absence of any commercial or financial relationships that could be construed as a potential conflict of interest. The reviewer MS and handling Editor declared their shared affiliation.
